# Canagliflozin potentially promotes renal protection against glycerol-induced acute kidney injury by activating the AMPK/SIRT1/FOXO-3a/PGC-1α and Nrf2/HO-1 pathways

**DOI:** 10.1007/s00210-025-04017-x

**Published:** 2025-04-21

**Authors:** Abeer Bishr, Ahmed M. Atwa, Bassant M. El-Mokadem, Mahmoud Nour El-Din

**Affiliations:** 1 Department of Pharmacology and Toxicology, Faculty of Pharmacy, Ahram Canadian University, Giza, Egypt; 2 Department of Pharmacology and Toxicology, Egyptian Russian University, Cairo, Egypt; 3 Department of Pharmacology and Toxicology, Faculty of Pharmacy, Egyptian Chinese University, Cairo, Egypt; 4 Department of Pharmacology and Toxicology, Faculty of Pharmacy, University of Sadat City, Menoufia, Egypt; 5 College of Pharmacy, Al-Ayen Iraqi University, AUIQ, An Nasiriyah, Iraq

**Keywords:** Acute kidney injury, Canagliflozin, Glycerol, PGC-1α, AMPK/SIRT1/FOXO-3a, Nrf-2/HO-1

## Abstract

The reno-protective potential of canagliflozin (Cana), an inhibitor of the sodium glucose–linked co-transporter-2 (SGLT-2), has been demonstrated in different models of kidney injury. However, its potential role in preventing glycerol (Gly)-induced acute kidney injury (AKI) remains to be divulged. Therefore, the aim of this study is to investigate the potential reno-protective effect of Cana and its underlying mechanism in a rat model of Gly-induced AKI. Rats were randomly allocated into five groups: normal, Gly, Gly pretreated with 10 mg/kg Cana, Gly pretreated with Cana 25 mg/kg, and normal pretreated with Cana 25 mg/kg for 14 consecutive days. Pretreatment with Cana improved renal structure and enhanced kidney functions manifested by reducing serum creatinine and blood urea nitrogen, as well as renal contents of neutrophil gelatinase-associated lipocalin and kidney injury molecule. Moreover, Cana signified its anti-inflammatory effect by reducing the Gly-induced elevation in renal contents of nuclear factor-κB and interleuκin-6. Additionally, Cana augmented the defense enzymatic antioxidants superoxide dismutase (SOD), manganese-SOD, and heme oxygenase-1, besides increasing the protein expression of the antioxidant transcription factor nuclear factor erythroid 2–related factor 2 to point for its ability to correct redox balance. Cana also upregulated the protein expression of the 5′ adenosine monophosphate-activated protein kinase (AMPK), Sirtuin1 (SIRT1), Forkhead box protein O3 (FOXO-3a), and peroxisome proliferator-activated receptor-gamma coactivator 1α (PGC-1α), as well as the transcriptional activity of growth arrest and DNA damage–inducible protein alpha (GAAD45a). In conclusion, Cana demonstrated potentially novel reno-protective mechanisms and mitigated the consequences of AKI through its antioxidant and anti-inflammatory properties, partially by activating the AMPK/SIRT1/FOXO-3a/PGC-1α pathway.

## Introduction

Acute kidney injury (AKI) is an abrupt onset of impaired renal function, resulting in the accumulation of nitrogenous waste products in the body (Gill et al. 2005; Lusiana et al. 2021), but is reversible in most cases (Makris and Spanou 2016). Various etiological factors contribute to AKI, including ischemia-reperfusion injury, nephrotoxicity, and rhabdomyolysis (Makris and Spanou 2016), many of which can be experimentally replicated.

In this context, a single intramuscular injection of glycerol (Gly) effectively mimics the pathological condition of rhabdomyolysis, leading to a significant decrease in glomerular filtration rate and renal blood flow (Reis et al. 2019). In Gly-induced AKI, myoglobin exerts direct cytotoxic effects on renal tubules, leading to the generation of reactive oxygen species (ROS) and the enhancement of lipid peroxidation (Hebert et al. 2023). Additionally, the inflammatory response, characterized by the activation of nuclear factor kappa-light chain–enhancer of activated B cells (NF-κB), results in the production of inflammatory cytokines and interleukins, further exacerbating kidney damage (Zhang and Sun 2015).

Since overproduction of ROS is one pivotal player in the context of AKI, it was reported that activation of the nuclear factor erythroid 2-related factor 2/heme-oxygenase-1 (Nrf-2/HO-1) signal alleviates renal injury by plummeting oxidative stress and inflammation in different kidney insults (Tong and Zhou 2017; Wu et al. 2017; Saha et al. 2020).

Amelioration of oxidative stress and inflammatory response has been mediated by the activation of different trajectories. One of the beneficial signals is the 5′ adenosine monophosphate-activated protein kinase (AMPK)/Sirtuin-1 (SIRT-1)/forkhead box protein O3 (FOXO-3a)/peroxisome proliferator–activated receptor-gamma coactivator 1α (PGC-1α). In this trail, AMPK serves as a key regulator of cellular energy status, responding to energetic stress induced by oxidative stress and inflammation (Hardie et al. 2012). Additionally, it modulates the activity of its downstream target, SIRT-1, which acts as an intracellular energy sensor by increasing cellular nicotinamide adenine dinucleotide^+^ (NAD^+^) levels (Giovannini and Bianchi 2017). Consequently, NAD^+^-dependent SIRT-1 influences the deacetylation of target proteins such as FOXO-3a (Ren et al. 2020), which promotes the expression of antioxidant genes, free radical scavengers, and manganese superoxide dismutase (MnSOD) (Meng et al., 2020). Moreover, AMPK and SIRT-1 are interconnected and jointly regulate PGC-1α (Cui et al. 2022), which is essential for mitochondrial biogenesis, oxidative phosphorylation, and adaptive thermogenesis (Liang and Ward 2006). Despite the proven beneficial role of this signaling pathway, its reno-protective effect was only recently verified in models of hyperglycemia-induced kidney injury (Park et al. 2020) and subsequently in a model of chronic kidney disease (Li et al. 2022).

Canagliflozin (Cana) is classified as a sodium-glucose-linked co-transporter (SGLT)−2 inhibitor primarily used in clinical practice for its antidiabetic properties (Plosker 2014). Beyond its established role in diabetes management, Cana has demonstrated additional benefits, including renal protection in both human studies (Kuriyama 2019) and animal models (Hasan et al. 2020a, b). Increasing evidence supports the anti-inflammatory and antioxidant effects of Cana, which contribute to reducing arterial stiffness, systemic blood pressure, and pathological remodeling of the heart and kidneys (Jakher et al. 2019; Hasan et al. 2020a, b).

Since effective therapy for AKI remains elusive, treatment strategies focus on minimizing damage to surviving nephrons and providing supportive care until kidney function is restored (Amery et al. 2015). Thus, our hypothesis posits that Cana may confer renoprotection against Gly-induced AKI through its antioxidant and anti-inflammatory mechanisms. Additionally, we aim to investigate the potential impact of the AMPK/SIRT1/FOXO3a/PGC-1α pathway in this context.

## Materials and methods

Male Wistar Rats weighing 150–200 g were obtained from Ahram Canadian University’s Faculty of Pharmacy’s animal house (ACU; Giza, Egypt). They were housed in a hygienic and well-supervised environment. Throughout the research, nourishment was provided ad libitum. The National Institutes of Health's Guide for the Care and Use of Laboratory Animals was followed while caring for them (NIH publication No. 85-23, updated 2011). This study was also approved by the Faculty of Pharmacy’s Research Ethics Committee (permission number: PO 121).

### Drugs and agents

Cana (Sigma-Aldrich, USA) was dissolved in hydroxypropyl methylcellulose (HPMC) (Lanxess, India) (Han et al., 2015), whereas Gly (El Nasr pharmaceutical company, Egypt) was used to cause renal damage.

### Induction of AKI

AKI in rats was induced by using a single dose of hypertonic Gly solution (50% v/v in normal saline) following 24 h of dehydration. The rats received an injection of this solution (10 ml/kg, i.m.) or equal volume of saline for rats of the normal group. The injected volume was divided equally between the two hind limbs.

### Experimental design and sampling

Thirty male rats were randomly divided into five groups. The first group received oral HPMC (0.5%) for 14 consecutive days as well as i.m. injection of saline on the 12th day and was considered the normal group. The second group also received oral HPMC (0.5%) for 14 consecutive days and then was injected with a single dose of Gly solution (10 ml/kg, i.m.) on the 12th day and served as Gly group (Wu et al. 2017). Rats of the third and fourth groups received Cana (10 mg/kg in 0.5% HPMC; p.o.) (Ali et al. 2019) and Cana (25 mg/kg in 0.5% HPMC; p.o.) (Ali et al. 2019), daily for 14 days, respectively, then were injected with Gly as in the second group and were considered as Gly + Cana 10 and Gly + Cana 25 groups, correspondingly. The latter group received Cana (25 mg/kg in 0.5% HPMC; p.o.) only and served as Cana 25 group. Rats from the second to the fourth groups were deprived of drinking water for 24 h before Gly administration. At the end of the investigation, on day 14, after 1 h of the last drug administration, rats were benumbed using ether. Blood samples were obtained from the femoral vein then centrifuged at 4000 rpm for 10 min. The isolated serum was used to estimate renal function parameters (creatinine and BUN). Directly after blood collection, animals were then sacrificed under anesthesia through cervical dislocation. The two kidneys were rapidly extracted, rinsed with saline, and dried among two filter papers after scarification. The left kidneys of three rats per group were fixed in 10% neutral buffer formalin for histopathological/immunohistochemical investigations, while the remaining three kidneys were split into two sections. The first half was kept in RIPA buffer with a protease inhibitor for western blot analysis, while the second part was stored in an RNA lysis solution for gene expression study using real-time PCR (RT-PCR). Other parameters were determined via ELISA technique by homogenizing the right kidneys of all rats in each group in ice-cold saline.

### Renal function tests

Creatinine and blood urea nitrogen were assessed colorimetrically using creatinine and urea biodiagnostic kits supplied by the Egyptian company of biotechnology (Cairo, Egypt).

### ELISA technique

The renal homogenate was used to determine the following parameters with ELISA kits: KIM-1 (Cat# RKM29-K01, Eagle Biosciences, NH, USA), NGAL (Cat# SEB388Ra, Cloud-Clone Corp, TX, USA), NF-κB (Cat# SEB824Mu, Cloud-Clone Corp, TX, USA), IL-6 ( Cat# SEA079Ra, Cloud-Clone Corp, TX, USA), HO-1 (Cat# E4525-100, BioVision, CA, USA), SOD (Cat# K335-100, BioVision, CA, USA), and MnSOD (Cat# LS-F6964, LifeSpan Biosciences, WA, USA).

### RT-PCR

Total RNA was extracted from kidney tissue using the SV Total RNA Isolation method (Invitrogen Life Technologies, Scotland, UK). The purity and concentration of the RNA were determined spectrophotometrically at 260 and 280 nm. Reverse transcription of the extracted RNA into complementary DNA (cDNA) was performed according to the manufacturer’s instructions using an RT-PCR kit (Fermentas Life Science Co., LT, USA). GADD45a expression was determined using RT-PCR and QuantiTest SYBR GREEN. The Applied Biosystems 3.1 software was utilized to amplify and analyze cDNA (StepOne, CA, USA). The RT-PCR test was adjusted for annealing temperature using the primer sets. The sequences of primers were GADD45a: forward primer 5′-CCATAACTGTCGGCGTGT-3′ and reverse primer 5′-CGCAGGATGTTGATGTCGT-3′ and β-actin: forward primer 5′-TCCGTCGCCGGTCCACACCC-3′ and reverse primer 5′-TCACCAACTGGGACGATATG-3′. GADD45a’s relative expression was evaluated according to β-actin.

### Western blot analysis

Each homogenized tissue sample was incubated with the ready prep TM protein extraction kit (total protein) supplied by Bio-Rad Inc. Bradford test was used to determine total protein content. On a 4% SDS-polyacrylamide gel, 20 µg of protein per lane was separated. A 0.45-µm nitrocellulose membrane was then used to transfer the protein. After 1 h of blocking with TBST buffer and 3% BSA, the membranes were incubated overnight at 4 °C with primary monoclonal antibodies against AMPK (Cat#: 2532, Cell Signaling Technology, MA, USA), and Phospho-AMPK (Thr172) (Cat#: 2531, Cell Signaling Technology, MA, USA), SIRT1 (Cat#: 2310, Cell Signaling Technology, MA, USA), Phospho-SIRT1 (Ser47) (Cat#: 2314, Cell Signaling Technology, MA, USA), FOXO-3a (Cat#: 12829, Cell Signaling Technology, MA, USA), Phospho-FOXO-3a (Thr32) (Cat #: 9464, Cell Signaling Technology, MA, USA), and anti‐PGC-1α antibodies (Cat#: sc-518025 1:1000) were obtained from Santa Cruz Biotechnology (CA, USA). After washing the blots, they were incubated for 1 h at room temperature with suitable horseradish peroxidase-conjugated secondary antibodies. The chemiluminescent substrate (ClarityTM Western ECL substrate, Bio-Rad) was applied to the blot as recommended by the manufacturer, and the chemiluminescent signals were collected using a CCD camera-based imager. The band intensities of the target proteins were determined using image analysis software and represented as arbitrary units (AU) relative to the β-actin expression on the ChemiDoc MP imager.

### Renal histopathology

Autopsy samples were obtained from the left kidneys of three rats and preserved for 24 h in 10% neutral buffer formalin. All specimens were cleaned with tap water for half an hour and then dehydrated with ethyl alcohol. Subsequently, they were embedded in paraffin blocks and sectioned (4 µm thickness) using a microtome. Tissue slices were then collected on glass slides, deparaffinized, and stained with hematoxylin-eosin. Under the light microscope, these sections were inspected blindly for histopathological changes (Bancroft and Gamble 2002). Briefly, for each slide of the kidney, a damage score of 0 (normal) to 4 (severe) was assessed for four criteria, namely, necrosis of renal tubules/coagulative necrosis, inflammatory cell infiltrates, hyaline and granular casts, and tubular regeneration (cytoplasmic basophilia/karyomegaly/nuclear crowding/mitosis). Lesions were assigned the following comprehensive rating standards: 4 (severe lesions diffused in all inspected sections), 3 (moderate lesions disseminated in some inspected sections), 2 (mild lesions focally demonstrated in several examined sections), 1 (few lesions displayed in one investigated section), 0 (no lesions). Accordingly, a maximal overall damage score (ODS) for kidney sections (two sections for each animal) was computed to be 16.

### Renal immunohistopathology determination of Nrf-2

To determine the level of Nrf-2 expression in deparaffinized kidney sections, immunohistochemical staining was performed. The sections were incubated with a diluted (1:100) 1ry antibody specific for Nrf-2 (Cat#: FNab05855, Wuhan Fine Biotechnology, Wuhan, China). The immunological response was observed using diaminobenzidine tetrachloride (DAB; Sigma-Aldrich, MO, USA). The immune reactivity of Nrf-2 was measured using image analysis software by calculating the percent area expression in three randomly selected fields in each region and averaging it using image analysis software (Image J, Version 1.46a, NIH, Bethesda, MD, USA).

### Statistical analysis

For statistical analysis, GraphPad Prism version 8.0 (GraphPad Prism, CA, USA) was utilized. The data were presented as mean ± standard deviation (SD). One-way analysis of variance (ANOVA) was performed for statistical analysis, followed by Tukey’s post hoc test to compare groups. Using the Kruskal-Wallis test (one-way ANOVA) and the multiple-comparison test of Dunn, we performed a non-parametric analysis of the data using the median (min-max) representation. *p* Values less than 0.05 were considered significant.

## Results

All assessed parameters in the normal group and Cana 25 group were comparable, so the comparison was limited to the normal group.

### Canagliflozin improves renal impairment induced by intramuscular injection of Gly

**Fig. 1:**
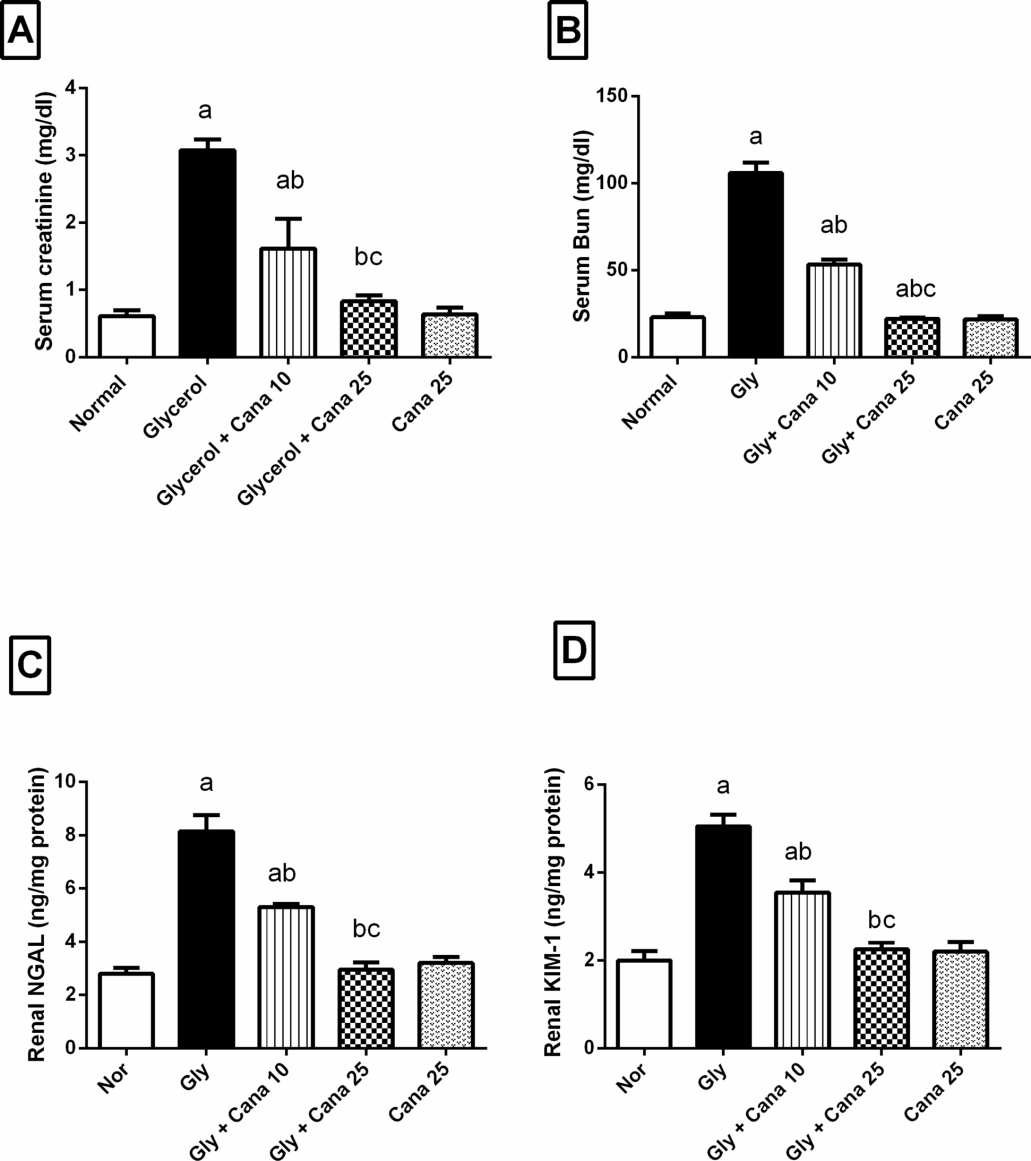
Effect of Cana (10 and 25 mg/kg) on **A** sCr, **B** BUN, **C** NGAL, and **D** KIM-1 levels, in Gly-induced AKI. Values are presented as means ± SD (*n* = 6). Statistical analysis was carried out using one-way ANOVA followed by Tukey’s post hoc test at *p* < 0.05. As compared to (a) nor, (b) Gly-group, (c) Gly + Cana 10. ANOVA, analysis of variance; Bun, blood urea nitrogen; Cana, canagliflozin; Gly, glycerol; KIM-1, kidney injury molecule-1; NGAL, neutrophil gelatinase associated lipocalin; Nor, Normal; sCr, serum creatinine

As illustrated in Fig. 1, Gly markedly boosted serum (A) sCr, (B) BUN, (C) NGAL, and (D) KIM-1 levels by 403%, 362%, 191%, and 153, respectively, in comparison to the normal control group. Nevertheless, pretreatment with Cana (10 mg/kg) significantly decreased the levels of sCr, BUN, NGAL and KIM-1 by 48%, 48%, 35%, and 30%, respectively, compared to Gly group. In comparison, pretreatment with the higher dose of Cana (25 mg/kg) dramatically decreased sCr, BUN, NGAL, and KIM-1 by 73%,79%, 64%, and 55%, respectively, as compared to Gly group.

### Canagliflozin altered the AKI-induced changes in NF-кB, IL-6 level, and SOD activity in Gly-treated rats

**Fig. 2:**
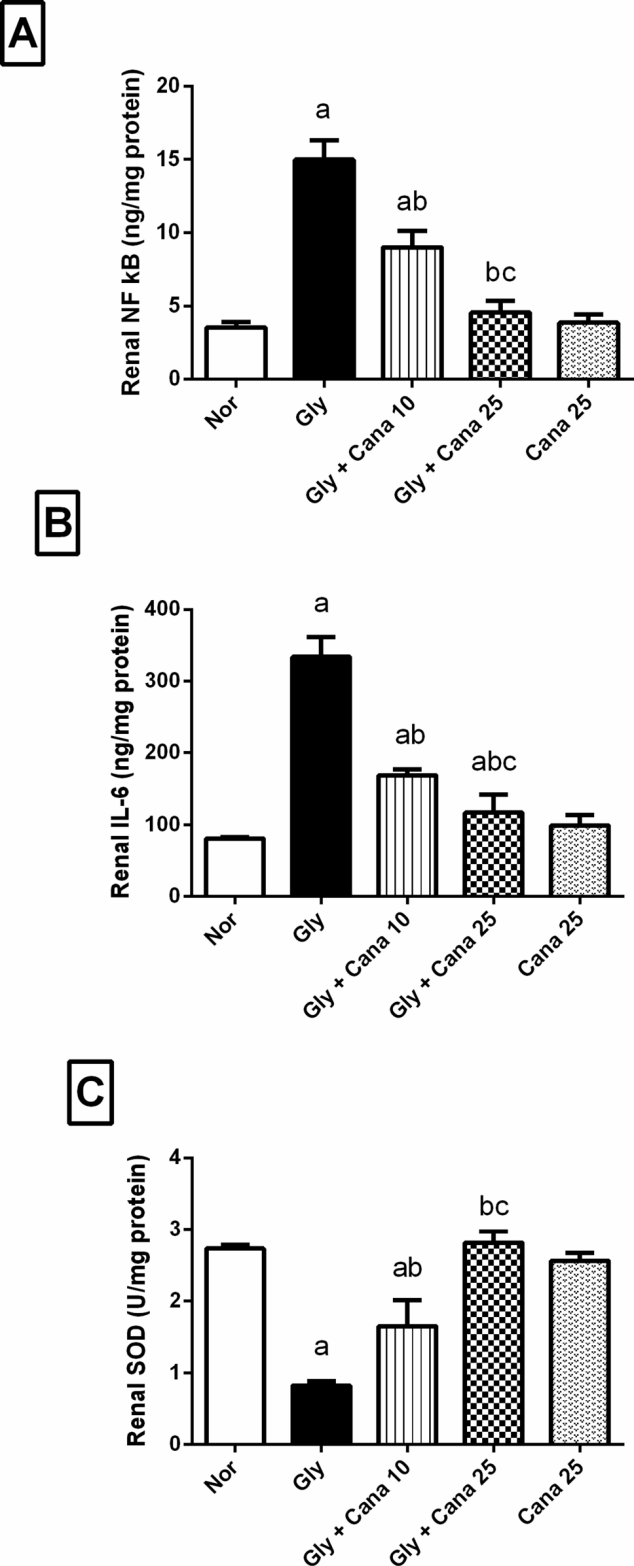
Effect of Cana (10 and 25 mg/kg) on renal **A** NF-kB, **B** IL-6 levels, and **C** SOD activity in Gly-treated rats. Values are presented as means ± SD (*n* = 6). Statistical analysis was carried out using one-way ANOVA followed by Tukey’s post hoc test at *p* < 0.05. As compared to (a) nor, (b) Gly-group, and (c) Gly + Cana 10. ANOVA, analysis of variance; Cana, canagliflozin; Gly, glycerol; IL-6, interleukin-6; NF-kB, nuclear factor kappa B; Nor, normal; SOD, superoxide dismutase

As found in Fig. 2, Gly injection significantly raised the levels of inflammatory markers (A) NF-кB (4.2-fold) and (B) IL-6 (4.1-fold) and reduced the antioxidant activity of (C) SOD by 70%, in comparison to the normal control group. On the other hand, pretreatment with Cana retuned the inflammatory markers and SOD in a dose-dependent manner. Nevertheless, administering Cana (10 mg/kg) greatly protected against Gly-mediated renal damage and dysfunction. This was evidenced by a notable reduction in NF-кB and IL-6 levels to 60% and 51%, respectively, and a substantial rise in SOD levels to 200% compared to the Gly group. In addition, Cana (25 mg/kg) significantly decreased the levels of inflammatory markers, with NF-кB lowered to 31% and IL-6 decreased to 35% compared to the Gly-induction group. In addition, Cana hindered the oxidative effect by nearly normalizing the SOD level compared to the normal control group showing significant increase to 342% compared to the Gly-induction group.

### Canagliflozin restores the level of HO-1 and MnSOD in Gly-treated rats

**Fig. 3:**
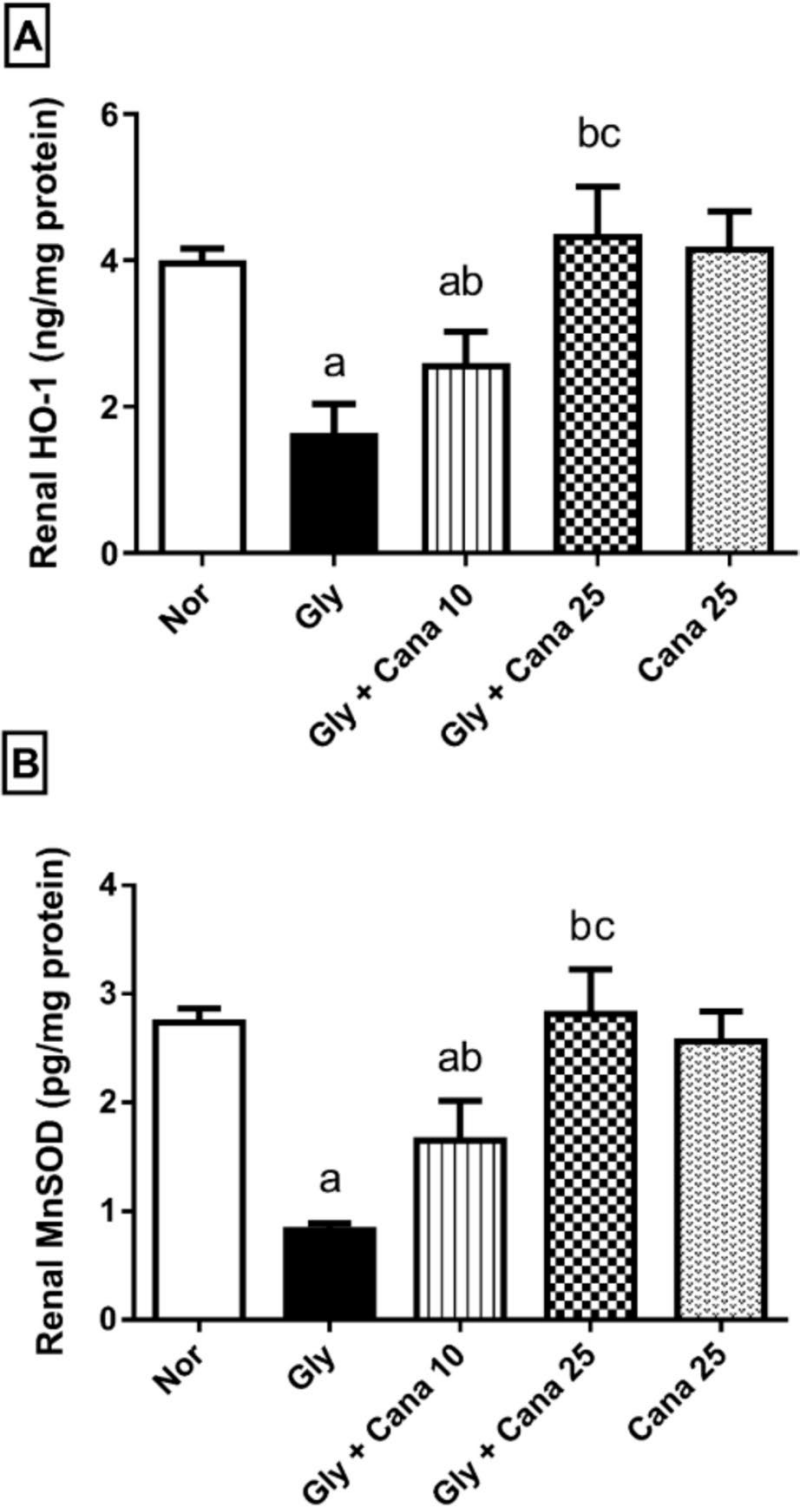
Effect of Cana (10 and 25 mg/kg) on **A** HO-1 and **B** MnSOD levels in Gly-induced AKI in rats. Values are presented as means ± SD (*n* = 6). Statistical analysis was carried out using one-way ANOVA followed by Tukey’s post hoc test at *p* < 0.05. As compared to (a) nor, (b) Gly-group, (c) Gly + Cana 10. ANOVA, analysis of variance; Cana, canagliflozin; Gly, glycerol; HO-1, Heme-oxygenase-1; MnSOD, manganese superoxide dismutase; Nor, normal group

In comparison to the normal group, Fig. 3 demonstrates severe oxidative stress in Gly-treated rats, as seen by a considerable fall in renal HO-1 (A) and MnSOD (B) levels to 41% and 30%, respectively. Meanwhile, pretreatment with Cana (10 mg/kg) reduced Gly-mediated oxidative damage by significantly elevating HO-1 and MnSOD in renal tissue by 158% and 200%, respectively, compared to the Gly-treated group. Cana (25 mg/kg) substantially reduced Gly-associated oxidative stress by restoring the normal levels of these antioxidant indicators, to 266% and 342%, respectively, compared to the glycerol group.

### Effect of Cana on mRNA expression of the stress-resistant gene, GADD45a

**Fig. 4:**
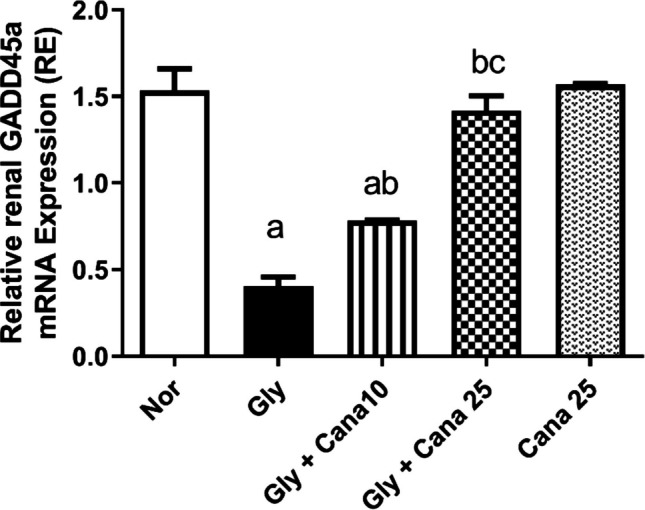
Effect of Cana (10 and 25 mg/kg) on mRNA expression of GADD45a using (RT-PCR) in kidney tissues. Gly decreased the expression of GADD45a significantly, while Cana was able to restore this reduction. Data presented as mean (relative expression) ± SD (*n* = 3). Statistical analysis was carried out using one-way ANOVA followed by Tukey’s post hoc test at *p* < 0.05. As compared to (a) nor, (b) Gly-group, (c) Gly + Cana 10. ANOVA, analysis of variance; Cana, canagliflozin; GADD45a, growth arrest and DNA damage-inducible protein alpha; Gly, glycerol; Nor, normal group; RT-PCR, real-time reverse transcription polymerase chain reaction

As shown in Fig.4, stress-resistant gene, GADD45a, was assessed using real-time reverse transcription-polymerase chain reaction (RT-PCR) to prove the protective effect of Cana. GADD45a expression was significantly reduced in the Gly-group compared to the normal group. Pretreatment with Cana boosted the level of GADD45a compared to the Gly-group. Noteworthy, Cana’s impact on GADD45a mRNA expression was dose-dependent as the high dose normalized its level.

### Canagliflozin restored the mitochondrial dynamics by activating phosphorylated AMPK/SIRT1/FOXO-3a/PGC-1α in Gly-treated rats

**Fig. 5:**
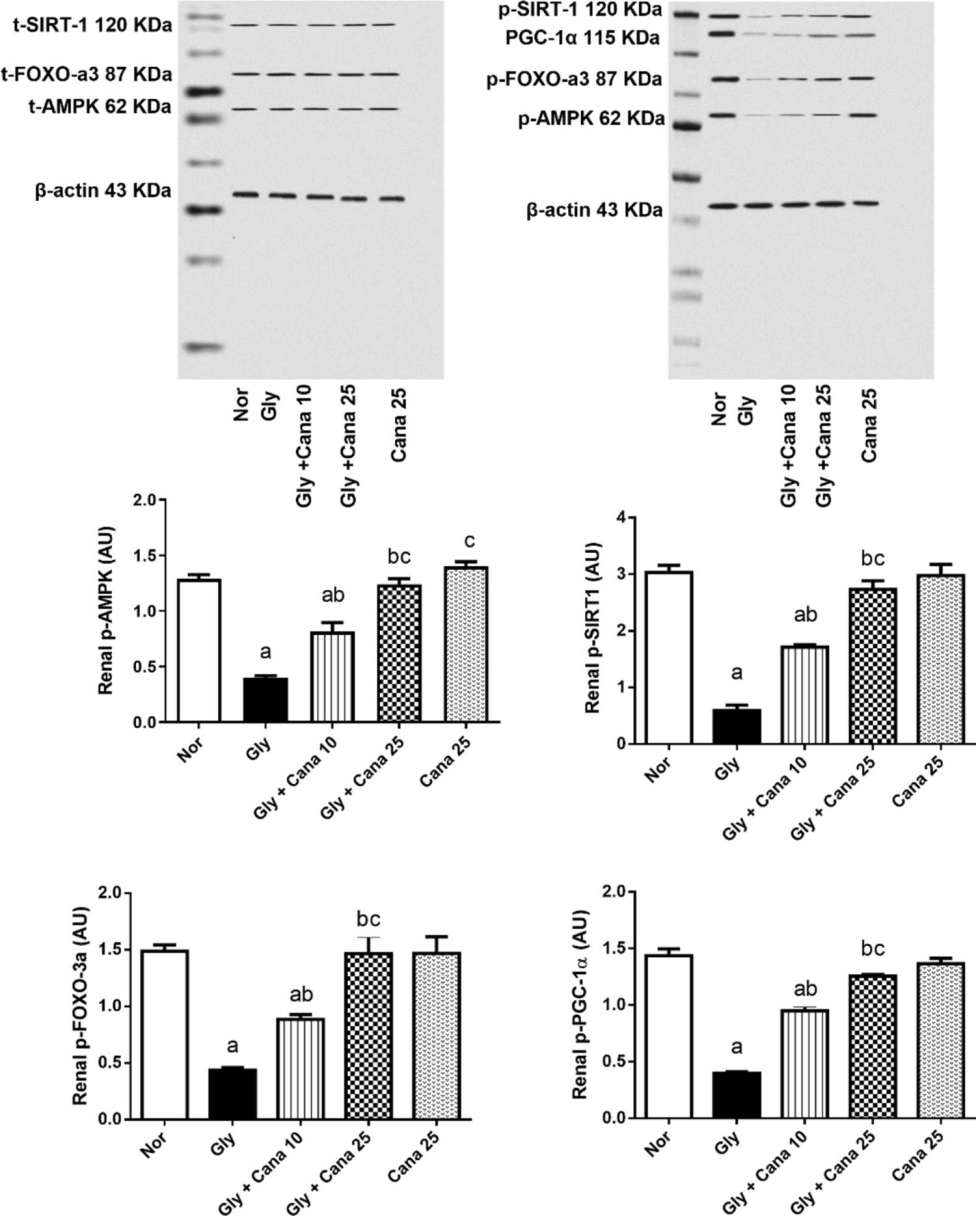
Effect of Cana (10 and 25 mg/kg) on phospho-AMPKα (**A**), phospho-SIRT1 (**B**), phospho-FOXO-3a (**C**), and phospho-PGC-1α (**D**) protein expressions in Gly-induced AKI in rats. Values are presented as means ± SD (*n* =3). Statistical analysis was carried out using one-way ANOVA followed by Tukey’s post hoc test at *p* < 0.05. As compared to (a) nor, (b) Gly-group, (c) Gly + Cana 10. AMPK, AMP-activated protein kinase; ANOVA, analysis of variance; AU, arbitrary unit; Cana, canagliflozin; FOXO-3a, forkhead box O3; Gly, glycerol; Nor, normal group; PGC-1α, peroxisome proliferator-activated receptor-γ coactivator; SIRT1, sirtuin-1

As presented in Fig. 5, relative to the normal group, administration of Gly caused a significant drop in the protein expression of renal (A) p-AMPK by 68%, (B) p-SIRT1 by 80%, (C) p-FOXO-3a by 70%, and (D) PGC-1α by 73%. Administration of Cana (10 mg/kg) markedly increased p-AMPK, p-SIRT1, p-FOXO-3a, and PGC-1α expression levels by 100%, 189%, 143%, and 143 %, respectively, as compared to the Gly group. Nevertheless, high dose of Cana (25 mg/kg) markedly increased p-AMPK, p-SIRT1, p-FOXO-3a, and PGC-1α expressions by 202%, 363%, 234%, and 223%, respectively, as compared to the glycerol group and brought them back to their typical values nearly similar to the normal group.

### Canagliflozin recuperates kidney oxidative injury induced by Gly by upregulating Nrf-2 expression

**Fig. 6:**
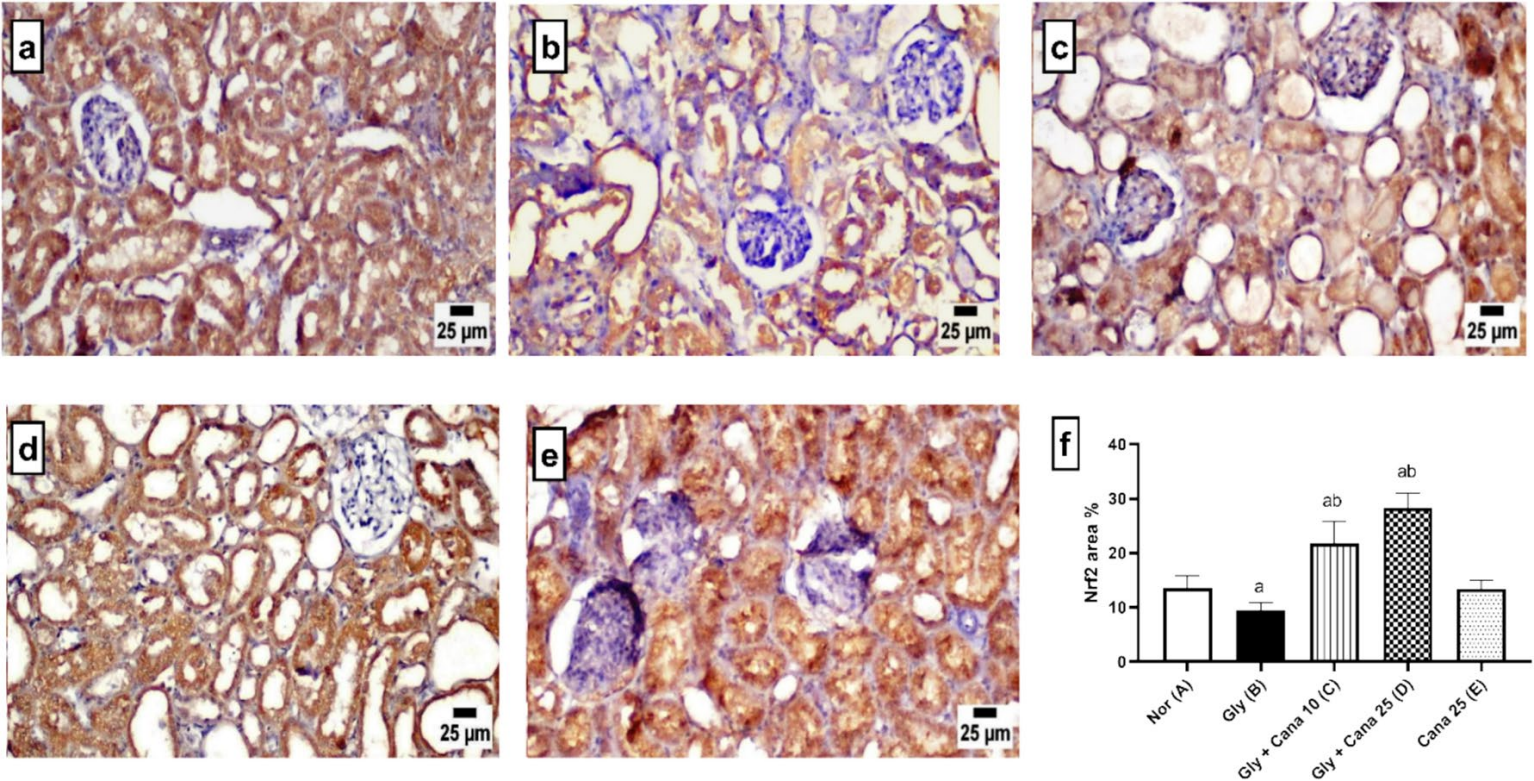
Effect of Cana (10 and 25 mg/kg) on Nrf-2 immunostaining expression of renal tubular epithelium. Immunostaining of kidney sections showing (a) Normal group; (b) Gly-induced AKI group; (c) Gly + Cana 10 group; (d) Gly + Cana 25 group; (e) Cana-only group resembled the normal group; and (f) chart depicts IHC area % expression of Nrf2 across groups (mean ± SE). *p < 0.05 vs. (a) normal, (b) Gly group. Cana, canagliflozin; Gly, glycerol; nor, normal

Our findings showed limited to moderate **Nrf-2** expression in the renal tubular epithelium of the normal group (Fig. 6a). The Gly group displayed lower **Nrf-2** expression in the degenerated and necrotic epithelial cells in the renal tubules (Fig. 6b). Higher expression was detected in low- and high-dose Cana-treated groups (Fig. 6c, d). Meanwhile, a significant higher level of **Nrf-2** expression was detected in the low dose–treated group compared to the Gly-group (Fig. 6c). The peak of expression was recorded in the high dose–treated group that exhibited a significant increase in **Nrf-2** expression in comparison with other experimental groups (Fig. 6d).

### Canagliflozin ameliorates histopathological abnormalities in Gly-induced AKI in rats

**Fig. 7:**
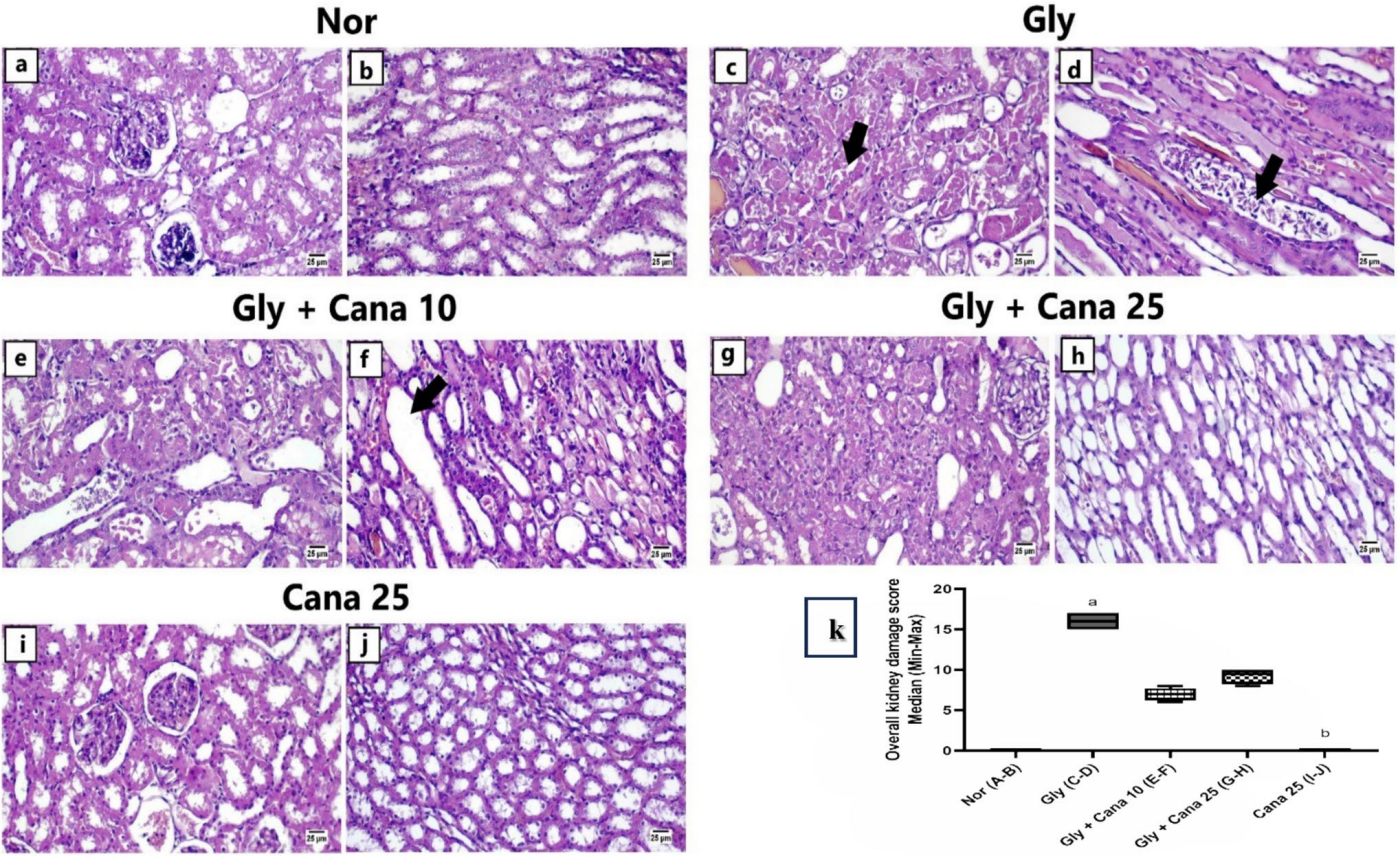
Kidney histological changes in Gly-induced AKI in rats using a light microscope. (a–j) Specimens were stained with H&E. (a) Normal control group showing normal renal cortex. (b) Normal control exhibiting normal renal medulla. (c) Gly-induced AKI showing extensive necrosis of the renal tubules in the renal cortex (arrow). (d) Gly-induced AKI showing higher power of sloughing of epithelial cells and necrotic debris into the tubular and ductal lumen in the renal medulla (arrow). (e) Pretreatment with Cana 10 mg/kg showing regenerative renal tubules along with necrotic tubules in the renal cortex. (f) Pretreatment with Cana 10 mg/kg showing cystic dilation in the renal medulla (arrow). (g) Gly + Cana 25 mg/kg displaying increased regenerative renal tubules the renal cortex. (h) Gly + Cana 25 mg/kg group showing apparently normal renal medulla. (i) Cana only–treated group showing normal renal cortex. (j) Cana only–treated group showing normal renal medulla. (k) The non-parametric data are presented as median (min-max) and statistically analyzed using the Kruskal-Wallis test followed by Dunn’s post hoc test at *p* < 0.05. As compared with (a) nor, (b) Gly group, (c) Gly + Cana 10 group, (d) Gly + Cana 25 group. Cana, canagliflozin; Gly, glycerol; nor, Normal group

Microscopic examination of kidney tissue (Fig. 7) from the normal control group (a and b) revealed the normal histology of both renal cortex and medulla; the renal cortex contained both glomeruli and renal tubules. The renal medulla showed both types of tubules and collecting ducts. Likewise, the Cana only group (i and j) showed histologically normal kidney tissues.

Administration of glycerol revealed serious histopathological alterations in the renal parenchyma of the induction group. The renal cortex showed extensive necrosis of renal tubules accompanied by accumulation of eosinophilic granular cast and necrotic debris in the tubular lumen. Multifocally, the renal tubules were filled with numerous red-orange hyaline casts admixed with cystic dilation of the adjacent renal tubules. The renal medulla showed widespread tubular casts resemble that found in the renal cortex and were associated with sloughing of epithelial lining into the ducts and tubular lumen (c and d).

The Cana 10 mg/kg treated group showed mild renal improvement characterized by a multifocal area of tubular regeneration that exhibited cytoplasmic basophilia, karyomegaly, and nuclear crowding along the affected tubules. Meanwhile, some individuals suffered from acute renal injury that showed necrotic renal tubules with desquamated epithelial cells and variable renal casts in the cortex and medulla (e and f).

The Cana 25 mg/kg treated group showed the greatest improvement. Multifocal areas of regenerative renal tubules were widespread in the renal cortex and medulla characterized by cytoplasmic basophilia along karyomegaly, nuclear crowding, and numerous mitoses. Some examined sections showed mild degeneration in few medullary renal tubules with apparently normal surrounding renal tubules and collecting ducts (g and h).

## Discussion

Glycerol (Gly) is one of the well-established paradigms for the induction of acute kidney injury (AKI) because of its myoglobin nephrotoxicity (Homsi et al. 2006). Rhabdomyolysis, which is the release of damaged cell components into the circulation, is a major part of the pathogenesis of Gly-induced AKI, resulting in inflammation, oxidative stress, and apoptosis (Li et al. 2019; Nara et al. 2016). Nearly 10% up to 50% of patients suffering from rhabdomyolysis have a certain degree of AKI with about 8% of the mortality rate (Korrapati et al. 2012). In our study, we determined the hindering of Gly-induced AKI through sodium glucose cotransporter-2 (SGLT2) inhibitor using canagliflozin (Cana). Also, we hypothesized the promising antioxidant and anti-inflammatory impact against renal damage. It has been reported previously the protective effects of Cana even in non-diabetic patients (Dekkers and Gansevoort 2020). As demonstrated before, AKI is characterized by the accumulation of nitrogenous wastes (Zheng et al. 2020). Our results supported this fact, as it was found that intramuscular administration of Gly caused a significant increase in both serum creatinine (SCr) and blood urea nitrogen (BUN) levels compared with the normal control animals, as earlier reported by Atwa et al. (2016). Similarly, a decline in kidney functions was proved by increasing renal tubular injury biomarkers such as neutrophil gelatinase-associated lipocalin (NGAL) and kidney injury molecule-1 (KIM-1); these results are consistent with Fauzi et al. (2016) and Li et al. (2019). The decline of renal functions in Gly-treated rats was opposed by Cana, as it significantly decreased BUN, sCr, NGAL, and KIM-1 levels, as shown by Abdelrahman et al. (2019) and Yale et al. (2013).

Concerning the histopathological examination herein, Gly produced considerable kidney impairment compared to the normal control group. It caused extensive necrosis of the renal cortex accompanied by the accumulation of eosinophilic granular cast and necrotic debris in the tubular lumen. Cana’s dose-dependent reno-protective effect was verified by enhancing Gly-induced histopathological alterations. Low-dose Cana showed mild renal improvement with a multifocal area of tubular regeneration. Meanwhile, the high dose exhibited the most remarkable improvement, as multifocal areas of regenerated renal tubules were widespread in the cortex and medulla of the kidney. These conclusions agree with the previous study, which used Cana in two doses (10 mg/kg and 30 mg/kg) to protect against cisplatin-induced nephrotoxicity (Abdelrahman et al. 2019). The current study showed that Cana mitigates the oxidative damage and inflammation caused by Gly, through the 5′ adenosine monophosphate–activated protein kinase/Sirtuin1/forkhead box protein O_3_/peroxisome proliferator–activated receptor-gamma coactivator 1α (AMPK/SIRT1/FOXO-3a/PGC-1α) axis in the kidney.

Furthermore, myoglobin released in response to Gly injection causes renal tubular injury through heme-iron–mediated lipid peroxidation (Nara et al. 2016; Singh et al. 2004). We validated the prevalence of oxidative stress in our investigation by observing a significant decrease in sodium dismutase (SOD) activity in the kidney after Gly administration. These results are in correlation with Adedapo et al. (2021). On the other side, our study showed that Cana has the ability to oppose the Gly-induced oxidative stress that was proven by increasing SOD levels. It has been evidenced previously that the increasing effect of Cana on SOD is cardiotoxic (Shi et al. 2021).

Deeply, both AMPK and SIRT1 synchronize with each other to regulate mitochondrial functions and inflammation (Ruderman et al. 2010). AMPK is a serine/threonine-protein kinase that acts as an intracellular energy sensor for cellular growth, autophagy, and metabolism (Glosse and Föller 2018; Kim et al. 2016). While SIRT1 is a NAD^+^-dependent deacetylase that acts on histone to remove the acetyl group (Nasrin et al. 2009). AMPK and SIRT1 connected to each other as SIRT1 deacetylation targets liver kinase B1, which triggers phosphorylation of AMPK (Wu et al. 2022). In turn, AMPK mediates the activation of SIRT1 through an increment of NAD^+^/NADH ratio (Potenza et al. 2019). The present study confirmed this relation, as it was found that Cana restored the reduction in the expression of AMPK and SIRT1 caused by Gly. Intriguingly, AMPK and SIRT1 influence various transcription factors and co-activators involved in gene expression regulation, such as FOXO-3a and PGC-1α, hence reducing inflammation, oxidative stress, and apoptosis (Tian et al. 2019). PGC-1α protects endothelium cells against ROS-induced endothelial dysfunction and cell death (Olmos et al. 2013). The FOXO-3a protein family is involved in various cellular processes and plays a crucial role in regulating antioxidant genes, and this regulation is controlled through the interaction with PGC-1α (Olmos et al. 2013). One of the main features of Gly-induced renal injury is the elevation of inflammatory cytokines such as nuclear factor-кB (NF-κB) and interleukin-6 (IL-6) (Saifulah et al. 2020). On the other side, Cana is verified to activate the AMPK/SIRT1 axis in addition to its antidiabetic actions (Yang et al. 2020). Alongside, it has been demonstrated previously the depressed actions of NF-κB and IL-6 through AMPK/SIRT1 in psoriasis and neurotoxicity (Lu et al. 2010; Xu et al. 2019b). NF-κB activation depends on acetylation of the p65 subunit through SIRT1 deacetylation (Xu et al. 2019a). Consistent with earlier data, this study demonstrated a Cana dose-dependent drop in NF-κB levels, followed by a decrease in the expression of IL-6.

Moreover, besides the protective actions of the AMPK/SIRT1 axis against Gly-induced renal injury, it is revealed that the phosphorylation of PGC-1α directly controls the lipid metabolism and mitochondrial genes (Lee 2010; Tian et al. 2019). PGC-1α has a major role in regulating mitochondrial remodeling and biogenesis (Wu et al. 1999). Our results presented the synchronization between AMPK/SIRT1 and PGC-1α, where all diminished with Gly-renal damage and raised up with Cana treatment. In addition, AMPK plays a vital role in activating FOXO-3a through phosphorylation, whereas SIRT1 promotes FOXO-3a deacetylation (Wang et al. 2017). Our results showed the Cana renoprotection against Gly via activation of the AMPK/SIRT1/FOXO-3a/PGC-1α axis. This is in correlation with earlier studies that proved Cana protection either through AMPK/SIRT1/PGC-1α or SIRT1/FOXO-3a in mitochondrial modeling and neurotoxicity, respectively (Hassanein et al. 2023; Yang et al. 2020). Under the control of SIRT1, FOXO-3a boosts its effect as a direct transcriptional regulator of antioxidant genes such as MnSOD in endothelial cells (Jacobs et al. 2008), and this regulation is also dependent on PGC-1α (Olmos et al. 2009). Furthermore, the present study found a dramatic uprise in MnSOD levels, which is linked to the increase of FOXO-3a and PGC-1α. Cana-treated rats showed a significant increase in the protein expression of the stress-resistant gene called GADD45a. This increment may be attributed to the deacetylation of FOXO-3a, as previous research confirmed the relationship between the deacetylation of FOXO-3a and the improvement of GADD45a expression (Wang et al. 2017). Moreover, Cana was able to replenish the decrease in SOD activity caused by Gly through the activation of SIRT1 (Z. Ren et al. 2019). The increase in SOD activity demonstrated that Cana has a dose-dependent antioxidant effect, and this finding aligns with prior results reported by Hasan et al. 2020a, b. The nuclear factor erythroid 2-related factor2/hemeoxygenase-1 (**Nrf-2**/HO-1) signaling pathway is also one of the investigated axes to prove the renoprotection of Cana. It has been evidenced the importance of **Nrf-2** and its regulated gene HO-1 in protection from oxidative stress (Loboda et al. 2016). The present study showed that, compared to a normal control group, **Nrf-2** and HO-1 expressions were dramatically decreased in Gly-treated rats, whereas they were elevated with Cana treatment in a dose-dependent manner. This increase in **Nrf-2** expression is probably controlled by PGC-1α, as mentioned before that PGC-1α/Nrf2 signaling attenuated the inflammatory response and restored kidney damage in septic acute kidney injury (Fan et al. 2024).

## Conclusion

In conclusion, for the first time, our research established that Cana in two different doses has a reno-protective impact against Gly-induced AKI by mitigating inflammation and oxidative stress via the activation of AMPK/SIRT1/FOXO-3a/PGC-1α and Nrf-2/HO-1 pathways. Besides, Cana supports its anti-inflammatory effect through hampering of NF-κB and IL-6. However, these findings are based on a short-term animal study; thus, further research is needed to confirm these effects in long-term studies and to explore additional underlying mechanisms

## Data Availability

All source data for this work (or generated in this study) are available upon reasonable request.
